# Leveraging electronic health records to improve management of noncommunicable diseases at primary healthcare centres in Saudi Arabia: a qualitative study

**DOI:** 10.1186/s12875-021-01456-2

**Published:** 2021-05-27

**Authors:** Ahmed Hazazi, Andrew Wilson

**Affiliations:** 1 Menzies Centre for Health Policy and Economics, Sydney School of Public Health, University of Sydney, Sydney, New South Wales Australia; 2 Department of Public Health, Faculty of Health Sciences, Saudi Electronic University, Riyadh, Saudi Arabia

**Keywords:** Electronic Health Record, Noncommunicable Diseases, Primary Healthcare Centres

## Abstract

**Background:**

Electronic Health Records (EHRs) can contribute to the earlier detection and better treatment of chronic diseases by improving accuracy and accessibility of patient data. The Saudi Ministry of Health (MOH) implemented an EHR system in all primary health care clinics (PHCs) as part of measures to improve their performance in managing chronic disease. This study examined the perspective of physicians on the current scope and content of NCDs management at PHCs including the contribution of the EHR system.

**Methods:**

Semi-structured interviews were conducted with 22 physicians working in chronic disease clinics at PHCs covering a range of locations and clinic sizes. The participants were selected based on their expertise using a combination of purposive and convenience sampling. The interviews were transcribed, analyzed and coded into the key themes.

**Results:**

Physicians indicated that the availability of the EHR helped organise their work and positively influenced NCDs patient encounters in their PHCs. They emphasised the multiple benefits of EHR in terms of efficiency, including the accuracy of patient documentation and the availability of patient information. Shortcomings identified included the lack of a patient portal to allow patients to access information about their health and lack of capacity to facilitate multi-disciplinary care for example through referral to allied health services. Access to the EHR was limited to MOH primary healthcare centres and clinicians noted that patients also received care in private clinics and hospitals.

**Conclusion:**

While well regarded by clinicians, the EHR system impact on patient care at chronic disease clinics is not being fully realised. Enabling patient access to their EHR would be help promote self-management, a core attribute of effective NCD management. Co-ordination of care is another core attribute and in the Saudi health system with multiple public and private providers, this may be substantially improved if the patients EHR was accessible wherever care was provided. There is also a need for enhanced capacity to support improving patient’s nutrition and physical activity.

## Background

Electronic health record (EHR) systems are increasingly viewed as a core tool in improving the overall quality, efficiency and safety of healthcare [1, 2]. In simple terms, adopting such systems enables healthcare professionals to provide better healthcare through better record keeping, decision making and patient care monitoring. At a system level EHRs facilitate monitoring of quality and outcome of health care and better identification of resource utilisation at a patient level. Research [3] stresses the potential of EHRs in healthcare facilities to improve system functioning in general and to reduce the total cost of healthcare services.

Saudi Arabia has made substantial progress in the implementation of EHRs in both hospitals and PHCs in recent years. The adoption of EHRs in Saudi Arabia is driven by the MOH's 2011 National E-Health Strategy, which was designed to facilitate the transition of the healthcare industry from paper-based to electronic platform in order to improve quality of healthcare services [4]. The MOH is the major government provider of 60% of healthcare services with a total of 282 hospitals and 2,361 primary health care facilities. Other quasi-governmental health institutions provide 10% while private sector provides 30% of healthcare services [5]. Previous studies on EHRs adoption have primarily focused on hospital settings rather than PHCs and reported lower rates of EHR adoption at PHCs [6, 7]. Those conducted in PHCs have not provided sufficient information about healthcare professionals' perceptions and NCDs management in relation to the implementation of EHRs in PHCs.

Primary healthcare centres (PHCs) play an important role in the diagnosis, management, and treatment of patients with non-communicable diseases (NCDs). The Saudi Ministry of Health (MOH) is implementing a national strategy to reduce the prevalence of chronic diseases including establishing chronic disease care clinics within PHCs [8]. The accuracy and accessibility of recording observations through EHRs contributes to the early detection and better treatment of chronic diseases. For this reason, the MOH decided to implement an EHR system in all PHCs in 2019 as part of measures to improve their performance in managing chronic disease, with the aim of reducing avoidable hospital presentation and admissions and improving overall healthcare services.

The results presented here are part of a mixed-methods study to examine the Saudi national policies and strategies to prevent and control NCDs and their risk factors. This paper provides an overview from the perspective of physicians of the current scope and content of NCDs management at PHCs including the contribution of the EHR system.

## Methods

A qualitative study using semi-structured interviews was undertaken with physicians who had at least one-year experience at PHCs to collect information about the current scope and content of NCDs management. The participants were selected based on their expertise and place of practice using a combination of purposive (targeting clinics in different communities) and convenience (doctors available and willing to participate at selected clinics) sampling [9]. A final sample of 22 physicians from the MOH working in chronic disease clinics at PHCs were interviewed covering a range of locations and clinic sizes in Riyadh, Saudi Arabi’s capital city. Recruitment ceased once data saturation of themes was achieved, meaning new additional information was no longer reported [10]. The first author (AH) conducted all the interviews using a semi-structured guide between May 2019 and August 2019.

The semi-structured interview guide was developed after reviewing the relevant literature on NCDs and healthcare systems in Saudi Arabia [11–14]. The physicians’ interview guide included questions regarding their views on the current EHR system, impact of EHR on management of NCDs and perceptions related to training in using the EHR. All physicians interviewed were willing to participate and responded to the interview questions. We ensured that there was no social desirability bias in this study as there was no prior relationship between the interviewees and interviewer. The interviews were audio recorded, transcribed and coded into key themes. A thematic analysis framework was used for the data analysis consisting of six steps: becoming familiar with the data, searching for themes, reviewing themes, defining and naming themes and writing the research report [15]. The use of thematic analysis helped to summarise key features of data and highlight similarities and differences in data sets. NVivo software was used to organise and code the data [16]. The approval to conduct this study was granted by the Ethics Committee of the Saudi Ministry of Health with a reference number. IRB log No:2019–0028 E.

## Results

Findings are grouped according to the following four themes that were derived from the interviews: current NCDs programs, impact on patient care, impact on physicians’ work and impact of EHR systems beyond the PHCs.

### Current NCDs programs

The Saudi MOH developed programs to reduce the incidence of NCDs and to improve the quality of life of the Saudi population. These programs are active in all government PHCs and focus on cardiovascular diseases, diabetes, respiratory disease, obesity and cancer (mainly breast and colon). The interviewees noted the value of the EHR to improving the effectiveness of these programs. The importance of the MOH in taking a leadership role in making these changes was identified as well as the need for ongoing training for physicians. The interviewees reported that that the MOH programs improved access to PHCs and the prevention components increased counselling about healthy eating habits and smoking cessation. However, they identified the need for more effective programs to promote healthy nutrition and physical activity including better access to dietitians/nutritionists. They stressed the importance of increasing outreach programs and widening participation in communities; they stated these goals required increased staff numbers at PHCs. They acknowledged that ongoing training had helped them to implement the MOH’s NCDs programs and to become familiar with using the EHR. They preferred online training to in-person training sessions, as this more flexible tailored training helped them to maintain their workflow efficiency. Finally, they emphasised the necessity for all physicians to train in use of the EHR and wanted advanced training on how to utilise the system’s customisation features and shortcuts in order to maximise their efficiency.

### Impact on patient care

The majority of physicians interviewed reported that the health information system’s adaptations and the availability of the EHR positively influenced encounters for patients with NCDs in their PHCs. The interviewees identified a number of key benefits of EHRs in their responses about caring for patients with NCDs, which can be broadly categorised as efficiency and useability. The physicians explained the ways in which the EHR made NCDs patient care more efficient. For example, one physician stated, ‘*Electronic health records make our work easier, as we get the patients’ records and results whenever the patients visit us – this saves time and effort*’ *(Phys 21, 11 years)*.

The *main theme* derived from the interviewees was the positive effects of EHR on the workflow of care. They emphasised the multiple benefits of EHR in terms of efficiency, including the accuracy of patient documentation and the availability of patient information, which were often difficult to find in their handwritten files. They also highlighted the benefits of having EHR data because it could help them to screen patients with NCDs for the early detection of comorbidities. As one physician stated, ‘*An important feature of the electronic file is the availability of the patients’ information that allows doctors to diagnose, treat and follow up more quickly’ (Phys 5, 8 years)*. In terms of useability, one interviewee highlighted that the ‘... *system is straightforward and easy to use’ (Phys 13, 4 years)*. However, the interviewee continued, the system lacks a patient portal and does not allow patients to access the full information about their health.

### Impact on physicians’ work

As part of the EHR, patients are required to book an appointment to visit an MOH PHC via ‘Mawid’, an electronic service that is a component of the EHR package established by the MOH [17]. Booking can be performed through the ‘Mawid’ application, by calling the MOH call centre or by visiting the patient’s PHC. The interviewees noted that these booking services helped them to organise their work – they were aware of what they would be doing for the day so that they could prepare, decrease wait times and easily plan for follow-up with patients. One participant described the importance of adopting EHR as follows: *‘... electronic health is a milestone in the history of healthcare in Saudi Arabia. It improves the management of chronic diseases, raises the quality of health services and reduces file errors’ (Phys 1, 12 years)*.

The interviewees also noted that EHR allows e-prescribe of medication, which is a huge improvement in the old system for accessing and prescribing medicine to NCDs patients. The e-prescription app, ‘Wasfaty’, was designed to facilitate the dispensing of medicine to patients by allowing them to receive the prescription electronically on their phones with a link to the nearest contracted pharmacy where they could obtain their prescriptions[18]. Wasfaty ensures the availability of medicines for NCDs patients. However, some of the interviewees reflected that they had received complaints from some of their elderly and disadvantaged patients, as they faced difficulties getting their medications. The interviewees also highlighted that Wasfaty is not built into the EHR system and operates on a different platform: *‘The current programs are separated and tiring to use, but their services are distinctive. I hope the MOH will combine all the programs into one platform’ (Phys 7, 5 years).*

### Linking of EHR systems

The interviewees stress the need to allow MOH primary healthcare centres and private clinics to share and link their systems to increase the functionality of EHR. The current EHR is only linked to MOH primary healthcare centres and does not connect to the private clinics and other quasi-governmental health institutions in Saudi Arabia. Interviewees reported issues when patients had previously attended quasi-governmental health institutions or private clinicians and then had follow up at other primary care facilities. One interviewee described this decentralised situation as follows: ‘*The challenges are the lack of information and not having the medical records and results from private sectors and other facilities outside the Ministry of Health’ (Phys 18, 3 years)*. The interviewees agreed that integrating patient records with other healthcare facilities would facilitate the exchange of health information between patients and healthcare professionals.

## Discussion

Our study examined the perspective of physicians on the current scope and content of NCDs management at PHCs including the contribution of the EHR system. Physicians in our study noted benefits of using EHR, which are supported in existing literature. They emphasised the multiple benefits of EHR in terms of efficiency, including the accuracy of patient documentation and the availability of patient information. These benefits have also been noted in other studies [19–21]. While interviewees recognized these benefits, shortcomings identified included the lack of a patient portal to allow patients to access information about their health and there is a need for the PHC EHR system to communicate or integrate across other private and governmental facilities.

### Patient portal

Accessing personal health records is seen as one of the most important tools for transforming a health information system [22]. This study found that physicians reported a very positive impact of the introduction of an EHR into PHCs. However, the impact of using the EHR in patient care at chronic disease clinics within PHCs is still probably not achieving its full potential. The current EHR does not allow patients full access to their health information. Current research on patient portals has shown that they can significantly improve patients’ adherence to screening recommendations and their ability to self-manage their NCDs by increasing their involvement in their health and focusing conversations on setting goals; overall this has improved patient-centred care delivery and the quality of care [22–25]. Other researchers have found that when patients with NCDs can access and view a patient portal, their satisfaction with care improves, allowing for better management of their conditions as well as increasing their empowerment and engagement in their own medical decisions [26–28].

To maximise the impact of care on NCDs patients, the EHR should promote a more patient-centric healthcare system, involve them in decision-making processes about their care and encourage them to modify unhealthy behaviours by monitoring indicators, introducing vital data and setting health goals. This allows patients to effectively participate in their own healthcare and increases the effectiveness of communication among physicians.

### NCDs programs

This research indicates that physicians saw a need for more effective programs to promote healthy nutrition and physical activity as well as better access to nutritionists. There is a great opportunity for multisectoral collaboration between nutritionists and dietitians in the private sector and MOH facilities. One option, which could be facilitated through the EHR, would be a nutrition referral scheme to facilitate formal referral of MOH PHC patients to an accredited dietitian. The interviewees acknowledged providing nutrition counselling forms part of their role as healthcare providers [29]; however, they are not always able to provide detailed nutrition advice that results in meaningful changes for their patients [30]; thus, collaboration between medical professionals and nutritionists is essential [31]. Studies have reported on the effectiveness and cost benefits of dietitians’ intervention in NCDs patients, including lowering risk factors associated with NCDs, blood pressure, glucose levels, lipid levels and weight; this is particularly effective when the dietitians are part of a multidisciplinary healthcare team [32–35].

The key enablers of increased physical activity among patients with NCDs in PHCs are social support, multi-disciplinary approaches and motivational interviewing [36]. Cost-effective interventions such as counselling based on self-reported activities can positively impact the health outcomes of NCDs patients, increasing levels of physical activity and reducing the risk of NCDs. Frank [37] found that physical activity interventions and counselling had a positive effect in the short- to medium-term on patients with NCDs. As these patients tend to regularly attend primary healthcare centres, screening programs for physical activity during consultations should be adopted. The EHR could help patients make positive *health behaviour changes* by tracking the delivery of preventive care that recommended across primary healthcare centres [38, 39] and montioring patient responses. This study stresses the need for interventions that encourage promoting the frequency of physical activity, for example, collaborating with gyms to create referral programs.

### System improvement

The MOH placed a lot of emphasis on technology to enhance NCD care. The Wasfaty prescribing program is considered a positive step, providing easier access for most patients to their medications. Improving medication policies and patient adherence reduces the economic and health burdens caused by NCDs [40]. However, Wasfaty may be less attractive to elderly patients living within walking distance to their PHCs, as they prefer to have their medications dispensed from the same PHC rather than being referred to a separate pharmacy. Solutions to this include policies that ensure prescribed medications are available at PHCs and home delivery/mail delivery. Overall, reducing barriers to obtaining medications improves adherence to medication [41]. Therefore, medication services need to be responsive to the needs of older and disadvantaged people.

The EHR has been implemented in chronic disease clinics at PHCs in order to improve the quality and efficiency of the healthcare they offer. The present study has found that physicians must use three different platforms to complete patient care actions, which increases their workload. A critical further improvement to the EHR functionalities is integrating the three platforms to simplify physician requirements in delivering clinical care. This is an important barrier to obtaining the full benefits of the EHR system, as reducing administrative task time and complexity can increase physicians’ clinical time, potentially quality of care and work satisfaction [42, 43].

### Integration of patient records

The MOH should prioritise completing linkages between the MOH and other private and governmental health agencies EHR systems. Disconnected EHR systems between sectors obviously have implications for efficiency of healthcare delivery. A singular system, or at least systems that can inter-communicate, should be implemented in all hospitals, clinics and specialised centres in the country to ensure one unified electronic patient record that is easily accessible regardless of where the patient is being treated. Integrating patient records could significantly reduce unnecessary duplication of services and care and positively impact the country’s healthcare budget, for example by preventing the unnecessary repetition of pathology and radiology tests [44]. It can reduce medication wastage and improves coordination and thereby quality of patient care by facilitating physicians access patient information where patients use different healthcare clinics. A structured exchange of clinical information among healthcare providers of NCDs patients enhances care coordination and improves that continuity and safety of care; it also supports better NCDs management [45, 46].

## Conclusion

EHRs have many positive benefits when applied in healthcare. The impact of Saudi EHR use on patient care at chronic disease clinics in PHCs is not being fully realised despite the positive attitudes of the interviewees in the present study. The need for the EHR system to allow patient access to their healthcare information was seen as a function which would enhance its capacity to support chronic disease management. Similarly, there is a need for the PHC EHR system to communicate or integrate across other private and governmental facilities. There is also a need for enhanced capacity to support patients with nutrition and physical activity. Overall efficiency of physician time would be enhanced by an integration of the EHR system with systems for patient booking, prescribing and referral. This research suggests future studies should monitor the impact of EHR utilisation on physician satisfaction, workload efficiency and patient care.

## Data Availability

The datasets generated and/or analysed during the current study are not publicly available due [containing interviews that analysed into themes] but are available from the corresponding author on reasonable request.

## References

[1] Graber ML, Byrne C, Johnston D (2017). The impact of electronic health records on diagnosis. Diagnosis.

[2] Schopf TR, Nedrebø B, Hufthammer KO, Daphu IK, Lærum H (2019). How well is the electronic health record supporting the clinical tasks of hospital physicians? A survey of physicians at three Norwegian hospitals. BMC Health Serv Res.

[3] Kruse CS, Kristof C, Jones B, Mitchell E, Martinez A (2016). Barriers to electronic health record adoption: a systematic literature review. J Med Syst.

[4] Ministry of Health. National E- Health Strategy 2011. Available from: https://www.moh.gov.sa/en/Ministry/nehs/Pages/Ehealth.aspx accessed 5 April 2021

[5] Ministry of Health. Annual statistical yearbook 2018. Available from: https://www.moh.gov.sa/en/Ministry/Statistics/book/Documents/ANNUAL-STATISTICAL-BOOK-1438H.pdf accessed 5 April 2021

[6] Jabali AK (2018). Electronic health records functionalities in Saudi Arabia: Obstacles and major challenges. Global J Health Sci.

[7] Reid Jr, ML. Adoption of electronic health record systems within primary care practices [Doctoral dissertation]. Walden University; 2018

[8] Ministry of Health. Chronic Diseases Clinics 2020 https://www.moh.gov.sa/Ministry/MediaCenter/News/Pages/NEWS-2007-2-17-007.aspx accessed 5 June 2020

[9] Creswell JW. A concise introduction to mixed methods research: SAGE publications; 2014.

[10] Guest G, Bunce A, Johnson L (2006). How many interviews are enough? An experiment with data saturation and variability. Field Methods.

[11] Hazazi AM, Chandramohan S (2017). Strengthening the Health Care System to Address the New Challenge of Non-Communicable Diseases in the Kingdom Of Saudi Arabia: A Systematic Review. International Journal of Scientific Study.

[12] Rahim HF, Sibai A, Khader Y, Hwalla N, Fadhil I, Alsiyabi H, Mataria A, Mendis S, Mokdad AH, Husseini A (2014). Non-communicable diseases in the Arab world. The Lancet.

[13] Khoja T, Rawaf S, Qidwai W, Rawaf D, Nanji K, Hamad A. Health care in Gulf Cooperation Council countries: a review of challenges and opportunities. Cureus. 2017 Aug;9(8). 10.7759/cureus.1586 PMC5650259 29062618

[14] Hassanain M. An overview of the performance improvement initiatives by the ministry of Health in the Kingdom of Saudi Arabia. INQUIRY: The Journal of Health Care Organization, Provision, and Financing. 2017 May 10;54:0046958017707872. 10.1177/0046958017707872 PMC5798699 28494624

[15] Braun V, Clarke V (2006). Using thematic analysis in psychology. Qual Res Psychol.

[16] QSR International Pty Ltd. NVivo 12 [computer software] 2020 [Available from: https://www.qsrinternational.com/nvivo-qualitative-data-analysis-software/home.

[17] Ministry of Health. *E-Services – (Mawid) 2020* https://www.moh.gov.sa/en/eServices/Pages/cassystem.aspx accessed 3 June 2020

[18] Wasfaty. About Wasfaty 2020 https://wasfaty.sa/ accessed 3 June 2020

[19] Kruse CS, Mileski M, Alaytsev V, Carol E, Williams A. Adoption factors associated with electronic health record among long-term care facilities: a systematic review. BMJ open. 2015 Jan 1;5(1). 10.1136/bmjopen-2014-006615 PMC4316426 25631311

[20] Sockolow PS, Bowles KH, Adelsberger MC, Chittams JL, Liao C (2014). Impact of homecare electronic health record on timeliness of clinical documentation, reimbursement, and patient outcomes. Appl Clin Inform.

[21] Mac McCullough J, Zimmerman FJ, Bell DS, Rodriguez HP (2014). Electronic health information exchange in underserved settings: examining initiatives in small physician practices & community health centers. BMC Health Serv Res.

[22] Wass S, Vimarlund V, Ros A (2019). Exploring patients’ perceptions of accessing electronic health records: Innovation in healthcare. Health Informatics J.

[23] Kruse CS, Argueta DA, Lopez L, Nair A (2015). Patient and provider attitudes toward the use of patient portals for the management of chronic disease: a systematic review. J Med Internet Res.

[24] Gray CS, Gill A, Khan AI, Hans PK, Kuluski K, Cott C (2016). The electronic patient reported outcome tool: testing usability and feasibility of a mobile app and portal to support care for patients with complex chronic disease and disability in primary care settings. JMIR Mhealth Uhealth.

[25] Coughlin SS, Prochaska JJ, Williams LB, Besenyi GM, Heboyan V, Goggans DS (2017). Patient web portals, disease management, and primary prevention. Risk management and healthcare policy.

[26] Urowitz S, Wiljer D, Dupak K, Kuehner Z, Leonard K, Lovrics E (2012). Improving diabetes management with a patient portal: Qualitative study of a diabetes self-management portal. J Med Internet Res.

[27] Goldzweig CL, Orshansky G, Paige NM, Towfigh AA, Haggstrom DA, Miake-Lye I (2013). Electronic patient portals: evidence on health outcomes, satisfaction, efficiency, and attitudes: a systematic review. Ann Intern Med.

[28] Woods SS, Schwartz E, Tuepker A, Press NA, Nazi KM, Turvey CL (2013). Patient experiences with full electronic access to health records and clinical notes through the My HealtheVet Personal Health Record Pilot: qualitative study. J Med Internet Res.

[29] Mitchell LJ, MacDonald-Wicks L, Capra S (2011). Nutrition advice in general practice: the role of general practitioners and practice nurses. Aust J Prim Health.

[30] Kent FM, Maddock BM, editors. Development of a Collaborative Care Curriculum. 2017 Australian and New Zealand Association for Health Professional Educators (ANZAHPE) Conference; 2017.

[31] Adamski M, Gibson S, Leech M, Truby H (2018). Are doctors nutritionists? What is the role of doctors in providing nutrition advice?. Nutr Bull.

[32] Al-Shookri A, Khor G, Chan Y, Loke S, Al-Maskari M (2012). Effectiveness of medical nutrition treatment delivered by dietitians on glycaemic outcomes and lipid profiles of Arab, Omani patients with type 2 diabetes. Diabet Med.

[33] Nisak MB, Ruzita A, Norimah A, Azmi KN (2013). Medical nutrition therapy administered by a dietitian yields favourable diabetes outcomes in individual with type 2 diabetes mellitus. Med J Malaysia.

[34] Parker AR, Byham-Gray L, Denmark R, Winkle PJ (2014). The effect of medical nutrition therapy by a registered dietitian nutritionist in patients with prediabetes participating in a randomized controlled clinical research trial. J Acad Nutr Diet.

[35] Sikand G, Cole RE, Handu D, deWaal D, Christaldi J, Johnson EQ (2018). Clinical and cost benefits of medical nutrition therapy by registered dietitian nutritionists for management of dyslipidemia: a systematic review and meta-analysis. J Clin Lipidol.

[36] Lion A, Vuillemin A, Thornton JS, Theisen D, Stranges S, Ward M (2019). Physical activity promotion in primary care: a Utopian quest?. Health Promot Int.

[37] Frank E, Segura C, Shen H, Oberg E (2010). Predictors of Canadian physicians’ prevention counseling practices. Can J Public Health.

[38] Gagnon M-P, Simonyan D, Godin G, Labrecque M, Ouimet M, Rousseau M (2016). Factors influencing electronic health record adoption by physicians: A multilevel analysis. Int J Inf Manage.

[39] De Leon SF, Shih SC. Tracking the delivery of prevention-oriented care among primary care providers who have adopted electronic health records. Journal of the American Medical Informatics Association. 2011;18(Supplement_1):i91-i5. 10.1136/amiajnl-2011-000219 PMC3241164 21856688

[40] National Center for Health Statistics. National health expenditures, average annual percent change, and percent distribution, by type of expenditure: United States, selected years 1960–2014. Hyattsville, MD: US Department of Health and Human Services, CDC, National Center for Health Statistics; 2015 (https://www.cdc.gov/nchs/data/hus/2015/094.pdf).

[41] Neiman AB, Ruppar T, Ho M, Garber L, Weidle PJ, Hong Y (2017). CDC grand rounds: improving medication adherence for chronic disease management—innovations and opportunities. MMWR Morb Mortal Wkly Rep.

[42] Meinert DB (2005). Resistance to Electronic Medical Records(EMRs): A Barrier to Improved Quality of Care. Informing Science: International Journal of an Emerging Transdiscipline.

[43] Ajami S, Bagheri-Tadi T (2013). Barriers for adopting electronic health records (EHRs) by physicians. Acta Informatica Medica.

[44] Anoushiravani AA, Patton J, Sayeed Z, El-Othmani MM, Saleh KJ (2016). Big data, big research: implementing population health-based research models and integrating care to reduce cost and improve outcomes. Orthopedic Clinics.

[45] Grover A, Joshi A (2015). An overview of chronic disease models: a systematic literature review. Global J Health Sci.

[46] Humphries C, Jaganathan S, Panniyammakal J, Singh S, Goenka S, Dorairaj P (2018). Investigating clinical handover and healthcare communication for outpatients with chronic disease in India: A mixed-methods study. PLoS ONE.

